# Emotion–tremor coupling in Wilson’s disease: EEG microstate C as a marker of salience network dysregulation

**DOI:** 10.1186/s13023-026-04427-x

**Published:** 2026-06-03

**Authors:** Pei-Zhu Zhang, Meng Wang, Xiao Wen, Pei Li, Ping Jin, Xin-Feng Ma, Kang Lin, Guang-an Tong, Gong-Qiang Wang, Yong-Zhu Han

**Affiliations:** 1 Institute of Neurology, Anhui University of Chinese Medicine, 357 Changjiang Middle Road, Hefei, 230061 China; 2 Department of Neurology, The Affiliated Hospital of Institute of Neurology, Anhui University of Chinese Medicine, Hefei, China; 3 Graduate School, Anhui University of Chinese Medicine, Hefei, China

**Keywords:** Wilson’s disease, Tremor variability, Embodied cognition, EEG microstates, Salience network

## Abstract

**Background:**

Emotional states are known to modulate tremor severity in Wilson’s disease (WD), but the neural mechanisms underlying this emotion–tremor coupling remain poorly understood. This study aimed to investigate the neurophysiological and structural substrates of emotion-induced tremor variability in WD patients using electroencephalography (EEG) microstate analysis, kinematic tremor tracking, and structural MRI.

**Methods:**

Forty-five tremor-dominant WD patients and 20 healthy controls underwent assessments with the Body Image Disturbance Questionnaire (BIDQ), Fahn-Tolosa-Marin Tremor Rating Scale (FTM-TRS), Self-Assessment Manikin (SAM), and Facial Expression Recognition (FER) tasks. Tremor kinematics were quantified via Kinovea, and EEG microstates were analyzed during emotion induction. Structural magnetic resonance imaging (MRI) was used to evaluate brain atrophy patterns.

**Results:**

WD patients exhibited greater social avoidance (*Z* = -5.721, *p* < 0.05), prolonged FER reaction times (1.78 s vs. 1.05 s, *p* < 0.001), and more negative face selections (6 vs. 5, *p* = 0.036). Negative emotional states were associated with significantly larger tremor amplitude (3.79 *px vs.* 3.16 *px*, *p* = 0.012). EEG microstates showed increased frequency of microstate C (4.74/min vs. 3.41/min, *p* < 0.001) and coverage (16.62% vs. 13.22%, *p* < 0.001) during negative emotion, which correlated with tremor severity (*ρ* = 0.319, *p* = 0.039). Regression analysis identified lentiform nucleus damage (*β* = 0.361), cerebellar atrophy (*β* = 0.300), and frontal atrophy (*β* = −0.386) as predictors of emotional arousal (*R²* = 0.277, *p* = 0.042).

**Conclusions:**

Emotion–tremor coupling in WD involves dysregulated salience networks and cerebellar-frontal-lentiform circuits, with EEG microstate C as a potential marker for targeted interventions.

## Introduction

Wilson’s disease (WD), an autosomal recessive disorder of copper metabolism (*ATP7B* gene mutation), leads to pathological copper accumulation in the liver and brain, particularly within the basal ganglia, cerebellum, and cortical regions [1, 2]. Neurological manifestations, such as tremor, dystonia, and dysarthria, occur in approximately 40–60% of patients and severely impair their quality of life [3]. Notably, clinical observations suggest that emotional states modulate tremor amplitude and frequency in WD patients [4, 5]; however, the neural mechanisms underlying this emotion–tremor coupling remain poorly understood.

Current pathophysiological models of WD underscore copper-induced neurotoxicity in the lentiform nucleus and cerebellar dentate nuclei—a process that disrupts the cortico-striato-thalamocortical (CSTC) circuits critical for motor control [6]. However, these models largely overlook the role of emotion-processing networks in motor symptom variability. Emerging evidence indicates that WD patients exhibit deficits in facial emotion recognition and heightened social anxiety [7], paralleling findings in functional movement disorders where negative emotions exacerbate tremor [8]. This raises a critical question — how do emotion-processing networks interact with motor circuits to drive tremor variability in WD? Dynamic systems theory posits that motor output emerges from the interaction between neural circuitry, body dynamics, and environmental constraints [9]. Within this framework, emotional states may act as “environmental constraints” that bias sensorimotor integration. Similarly, embodied cognition theory proposes that bodily states (e.g., tremor-induced proprioceptive feedback) directly shape emotional experiences, forming a closed loop between perception, emotion, and action [10]. For WD patients, chronic tremor may distort body schema representations (as measured by the Body Image Disturbance Questionnaire, BIDQ), amplifying emotional arousal and further destabilizing motor output — a hypothesis supported by recent magnetic resonance imaging (MRI) studies showing aberrant insula activation during emotional tasks in WD [11].

Electroencephalogram (EEG) microstates, which are transiently stable scalp field topographies (100–200 ms duration), provide a powerful tool to capture the rapid dynamics of large-scale brain networks [12]. Four canonical microstates (A-D) have been linked to distinct networks: auditory (A), visual (B), salience (C), and attentional (D) networks [13]. Notably, microstate C, associated with the anterior insula and anterior cingulate cortex, is modulated by emotional arousal and correlates with interoceptive awareness [14] a process likely disrupted in WD owing to basal ganglia-cerebellar pathology. Despite these advances, critical gaps persist: (1) No previous study has quantitatively examined how emotional states dynamically modulate tremor kinematics in WD using motion tracking. (2) The neurophysiological signatures of emotion-tremor interactions, particularly EEG microstate dynamics (salience network activity), have never been explored in WD. (3) The structural substrates (lentiform nucleus, cerebellum, frontal cortex) underlying emotion-motor integration in WD remain unidentified. To our knowledge, this is the first study to combine kinematic tremor analysis, EEG microstate dynamics, and structural MRI to investigate emotion-tremor coupling in WD.

This study bridges these critical gaps through an innovative multimodal approach, seamlessly integrating kinematic tremor analysis, EEG microstate examination, and structural MRI. We hypothesize that negative emotions are associated with increased tremor amplitude and dynamically alter EEG microstate activity in WD patients. By rigorously testing these hypotheses, we aim to unravel the complex spatiotemporal mechanisms underlying emotion-tremor coupling in WD, paving the way for novel neuromodulation therapy targets.

## Methods

### Participants

A total of 45 tremor-dominant WD patients (28 males, 17 females; age range: 17–55 years) were recruited from the Department of Neurology in Affiliated Hospital of Institute of Neurology of Anhui University of Chinese Medicine between August 2023 and June 2024. All patients met the following criteria: (1) confirmed WD diagnosis per the Leipzig criteria (clinical symptoms, Kayser-Fleischer rings, serum ceruloplasmin < 20 mg/dL, and/or elevated 24-hour urinary copper excretion) [15]; (2) postural/action tremor confirmed by neurologists using the Fahn-Tolosa-Marin Tremor Rating Scale (FTM-TRS) [16]; (3) All enrolled patients had not taken benzodiazepines, SSRIs, or antipsychotics within the two weeks prior to the assessment. The exclusion criteria were as follows: comorbid neuropsychiatric disorders (e.g., Parkinson’s disease, epilepsy), severe hepatic dysfunction (Child-Pugh class C), or inability to complete assessments.

Twenty age-, sex-, and education-matched healthy controls (HCs: 10 males, 10 females) were enrolled. The sample size was determined via G*Power 3.1 (α = 0.05, power = 0.80, effect size = 0.6 for tremor-emotion correlations), yielding a minimum requirement of 40 WD patients and 18 HCs.

### Clinical and behavioral assessments

#### Body image disturbance

The Body Image Disturbance Questionnaire (BIDQ): A validated 7-item Chinese version [17] was used to assess distress related to tremor (e.g., “How often do you avoid social situations due to tremor?“). Each item is scored from 0 to 8 (total range: 0–56; Cronbach’s α = 0.87).

#### Tremor severity

FTM-TRS: This method assesses tremor when the patient is at rest, changing position, or performing movements. Higher scores indicate more severe tremor symptoms. The scale rates tremor amplitude (0–4), frequency (0–4), and functional impact (0–4) across 9 tasks (total range: 0-144) .

#### Emotional processing

Self-Assessment Manikin (SAM): Participants rated emotional valence (1 = unpleasant to 9 = pleasant), arousal (1 = calm to 9 = excited), and dominance (1 = controlled to 9 = in control) after each emotion induction block [18].

#### Facial expression recognition (FER) task [19]

Using the Chinese Facial Affective Picture System (CFAPS), 24 images (8 happy, 8 neutral, and 8 fearful; balanced for gender) were displayed on a 24-inch LCD monitor (PsychoPy v2022.2.5). The participants categorized emotions via keypress (1 = positive, 2 = neutral, 3 = negative); reaction time (RT) and accuracy were recorded.

### Experimental paradigms

#### Emotion induction

Three 5-minute video blocks (positive, neutral, negative) from the SJTU Emotion EEG Dataset (SEED) [20] were used to induce the emotional experiences; these videos were validated to induce effective valence/arousal (positive: valence = 7.2 ± 0.8, arousal = 6.5 ± 1.1; negative: valence = 2.3 ± 0.6, arousal = 7.0 ± 1.3). After a resting-state EEG recording (eyes open, 5 min), an emotional video was presented. Each emotion block included 5 s of instruction (“Positive/Neutral/Negative video upcoming”), a 5-min video, a 45 s SAM rating, and a 30 s post-video tremor recording (Kinovea motion tracking). The order of emotion blocks was counterbalanced across participants.

#### Tremor kinematics

Participants held their arms outstretched (90^°^ shoulder abduction) with a 500 g wrist weight. A digital camera (Sony AX700, 120 Hz) recorded hand motion. Using Kinovea software for Windows (version 0.9.5) [21], the ulnar styloid process was tracked. Tremor amplitude (*px*) was calculated as the root mean square (RMS) of displacement over 30s, normalized to baseline.

### Neurophysiological and neuroimaging data acquisition

#### EEG recording and preprocessing

**Fig. 1:**
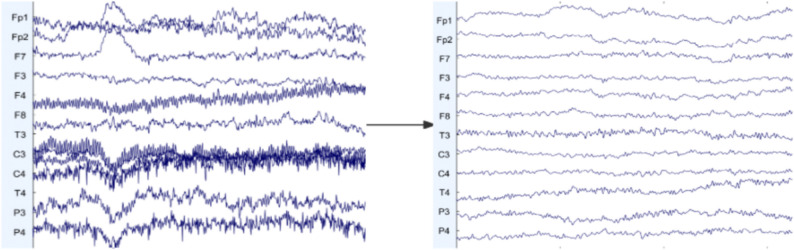
Schematic of EEG preprocessing. Raw EEG data were processed in MATLAB to remove artifacts from electromyography (EMG), electrooculography (EOG), and other sources

EEG data were acquired using a 32-channel EEG system (Brain Products ActiChamp, 500 Hz sampling, 0.1–100 Hz bandpass). A1 and A2 were used as reference electrodes. The electrode positions were placed in strict accordance with the international 10–20 system standard, and the impedance was kept below 10 kΩ. EEG data were preprocessed using EEGLAB v2024.0 [22]. Epochs were segmented into 30 s video-watching periods. Preprocessing steps included downsampling to 256 Hz, bandpass filtering (1–40 Hz), notch filtering (49–51 Hz), baseline correction, and independent component analysis (ICA) for removal of ocular and motion artifacts (Fig. 1). Electromyography (EMG) and electrooculography (EOG) artifacts were removed via ICA.

**Fig. 2:**
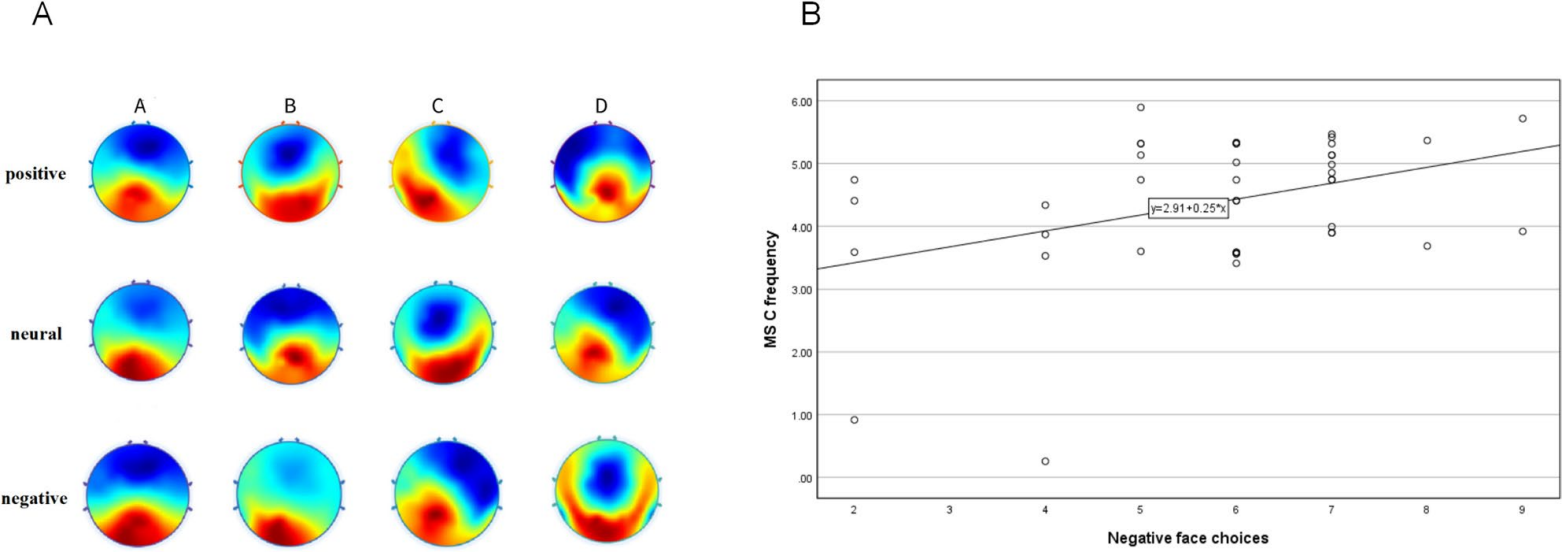
(**A**) Topographic maps of microstates **A**–**D**. **A**: Microstate **A**. **B**: Microstate **(B) C**: Microstate **(C) D**: Microstate (**D**) (**B**) Correlation between the frequency of Microstate C and negative facial selection. According to the Spearman analysis, the frequency of microstate C responses under negative emotions was positively correlated with the number of negative facial choices

**Fig. 3:**
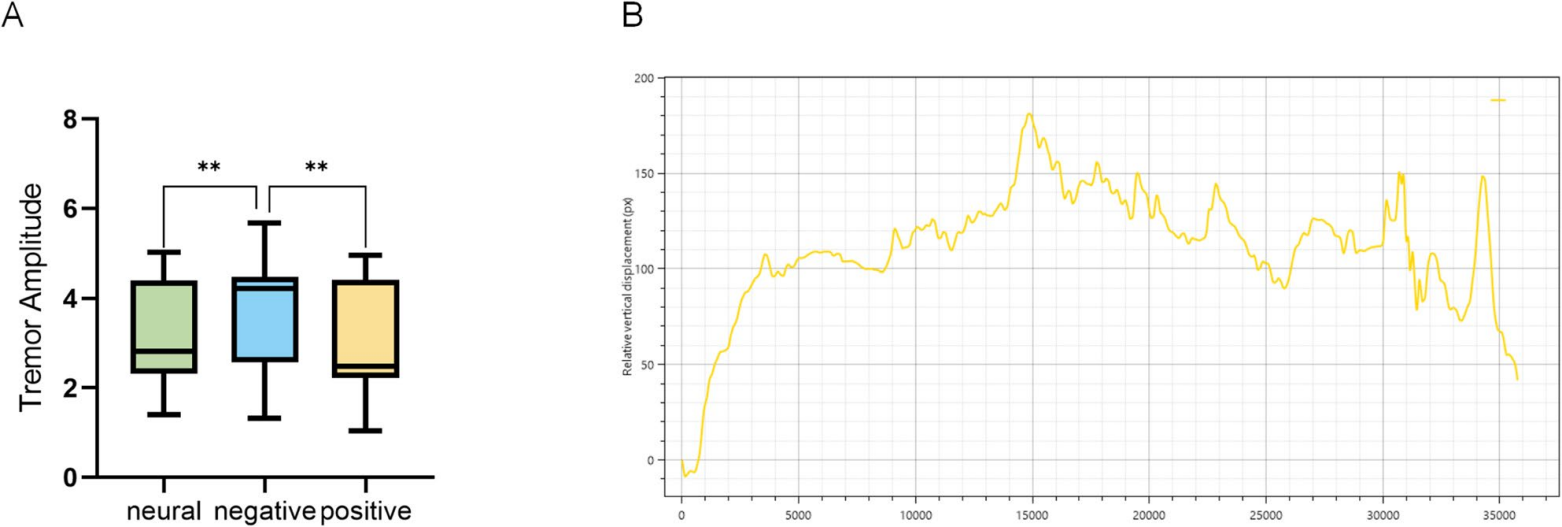
(**A**) Comparison of tremor amplitude in different emotion states. Light green: neural emotion state. Light blue: negative emotion state. Yellow: positive emotion state. There were differences in tremor amplitude between negative emotion and neutral and positive emotion conditions according to one-way ANOVA analysis. (**B**) Kinematic traces of tremor displacement during negative states. Kinovea software was used to track the motion of tremor videos and generated the trajectory of ulnar styloid process movement

**Fig. 4:**
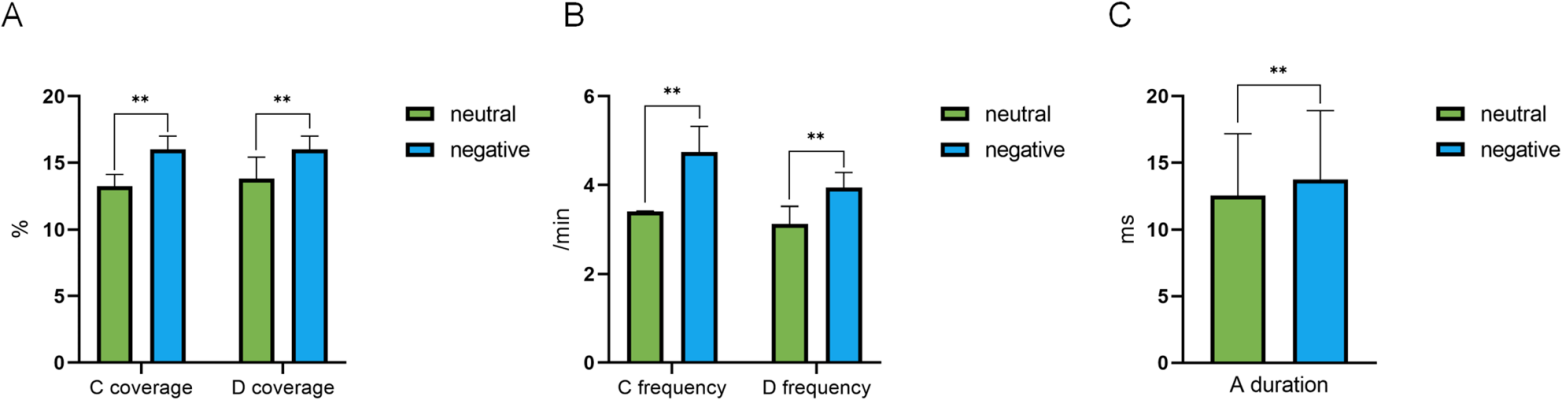
(**A**) Comparison of coverage in microstates **C** and **D**. According to the Mann–Whitney U tests, microstates **C** and **D** had higher coverage rates under negative emotions than under neutral emotions. (**B**) Comparison of frequency in microstates **C** and **D**. According to the Mann–Whitney U tests, microstates **C** and **D** had a faster frequency under negative emotion than under neutral emotion. (**C**) Comparison of duration in microstate **A**. According to the Mann–Whitney U tests, microstate A had a longer duration under negative emotion than under neutral emotion

#### EEG microstate analysis

*Clustering*: Microstates were extracted via K-means clustering (Cartool v3.8) [23]. Four canonical microstates (A-D) were identified across all participants (global explained variance > 80%). Parameters: For each microstate, frequency (number/min), duration (ms), and coverage (%) were computed (Fig. 2A).

#### Structural MRI

*Acquisition*: A 1.5T Siemens Magnetom Aera scanner (T1: TR = 2300 ms, TE = 2.98 ms; T2-FLAIR: TR = 9000 ms, TE = 97 ms; slice thickness = 5 mm) was used. *Analysis*: Two neuroradiologists blinded to the diagnoses assessed atrophy in the lentiform nucleus, cerebellum, and frontal cortex using visual rating scales (0 = normal to 3 = severe atrophy) [24].

### Statistical analysis

Two-sample t-tests, medians, and interquartile ranges were used to analyze the clinical and demographic variables. Chi-square tests and Wilcoxon signed-rank tests were employed for between-group comparisons. One-way ANOVA was used to analyze tremor amplitude variability. Structural predictors of SAM arousal were assessed via stepwise regression (entry *p* < 0.05, exit *p* > 0.10). Multiple comparisons were corrected using the Benjamini-Hochberg procedure (false discovery rate = 0.05) for microstate analyses. SPSS 25.0 was used for statistical analysis, and *p* values < 0.05 were considered significant.

### Ethical approval

The study protocol was approved by the Ethics Committee of Anhui University of Chinese Medicine (Approval No. 2023-19). All participants or their legal guardians provided written informed consent.

## Results

### Demographic and clinical characteristics

Tremor-dominant WD patients (*n* = 45) and healthy controls (HCs, *n* = 20) were well matched for age, sex, and education level (all *p* > 0.05). WD patients had a median disease duration of 6 years (IQR: 3.5–9.0) and moderate tremor severity (FTM-TRS total score: 25.6 ± 5.0), (Table 1).

**Table 1:** Demographic and clinical features

Variable	WD Group (*n* = 45)	HC Group (*n* = 20)	Statistics	*p*-Value
Age (years)	33.04 ± 7.016	31.80 ± 5.606	*T* = 0.699	0.487^a^
Sex (male/female)	45(27/18)	20(10/10)	*U* = -0.746	0.456^b^
Education (years)	8.0[8.0,11.0]	8.00 ± 3.026	*U* = -1.647	0.099^c^
Disease duration (years)	14.64 ± 8.70	–	–	–
FTM-TRS total score	25.6 ± 5.0	–	–	–

‘–’ indicates no score. ^a^:Two-sample T test. ^b^:Chi-square test. ^c^:Nonparametric Kruskal-Wallis test

### Body image disturbance and emotional processing

WD patients reported significantly greater body image distress (BIDQ total score: 25.6 ± 4.5 vs. 6.6 ± 2.7, *t* = 17.416, *p* < 0.001) and social avoidance (BIDQ items 3, 5, 6 and 7 scores, all *p* < 0.05). In the FER task, WD patients exhibited longer reaction times (1.78 s [1.20, 2.35] vs. 1.05 s [0.90, 1.30], *Z* = -4.735, *p* < 0.001) and selected negative faces more frequently (6.0 [5.0,8.0] vs. 5.0 [4.0,6.0], *Z* = -2.581, *p* = 0.036; Table 2).

**Table 2:** BIDQ and FER task performance

Measure	WD Group (*n* = 45)	HC Group (*n* = 20)	Statistics	*p*-Value
BIDQ total score	25.6 ± 4.5	6.6 ± 2.7	*t =* 17.416	< 0.001^a, b^
BIDQ3(distress)	3.0[1.0,4.0]	0.0[0.0,0.0]	*Z* = -5.872	< 0.001^a, c^
BIDQ5(Social impact)	3.0[1.0,4.0]	1.0[0.0,1.75]	*Z* = -3.716	< 0.001^a, c^
BIDQ6(Role function)	2.0[0.0,4.0]	0.0[0.0,1.75]	*Z* = -3.023	0.002^a, c^
BIDQ7(Avoidance)	5.0[3.0,6.0]	2.05 ± 1.504	*Z* = -5.721	< 0.001^a, c^
FER reaction time (s)	1.78[1.20,2.35]	1.05[0.90,1.30]	*Z* = -4.735	< 0.001^a, c^
Negative face choices	6.0[5.0,8.0]	5.0[4.0,6.0]	*Z* = -2.581	< 0.036^a, c^

^a^:*P* < 0.05 indicates a statistically significant difference. ^b^:Two-sample t-test. ^c^:Nonparametric Kruskal-Wallis test

### Emotion-induced tremor variability

One-way ANOVA revealed a significant main effect of emotional state on tremor amplitude (*F* (2,132) = 4.839, *p* = 0.009, *η*² = 0.052). Post-hoc Bonferroni tests revealed significantly larger tremor amplitude during negative emotion (3.79 ± 1.18 *px*) than during positive emotion (3.10 ± 1.22 *px*, *p* = 0.012) and at baseline (3.16 ± 1.05 *px*, *p* = 0.006), whereas no difference was found between baseline and positive emotion (*p* > 0.05), (Fig. 3A, B).

### EEG microstate parameters

Wilcoxon signed-rank tests were used to compare EEG microstate parameters between emotional conditions within the WD group. Compared with neutral emotion, negative emotion was associated with longer microstate A duration (13.72 ms vs. 12.52 ms, *Z* = 2.83, *p* = 0.005), higher microstate C frequency (4.74/min vs. 3.14/min, *Z* = 3.92, *p* < 0.001), greater microstate C coverage (16.62% vs. 13.22%, *Z* = 3.87, *p* < 0.001), higher microstate D frequency (3.94/min vs. 3.13/min, *Z* = 4.01, *p* < 0.001), and greater microstate D coverage (16.14% vs. 13.81%, *Z* = 4.15, *p* < 0.001). No significant differences were found between positive and neutral emotion for any microstate parameter (all *p* > 0.05), (Fig. 4A, C). In contrast, no significant differences were observed between positive emotion and neutral conditions for any microstate parameter (all *p* > 0.05), indicating that only negative emotion reliably modulated EEG microstate dynamics in WD patients.

### EEG microstate-behavior correlations

Microstate C frequency during negative emotion correlated with negative facial choices (*ρ* = 0.338, *p* = 0.029); microstate C coverage correlated with negative-emotion tremor amplitude (*ρ* = 0.319, *p* = 0.039). Microstate C coverage (*ρ* = 0.352, *p* = 0.022), frequency(*ρ* = 0.361, *p* = 0.019) and microstate D frequency (*ρ* = 0.353, *p* = 0.022) during positive emotion each correlated with positive emotion valence (Fig. 2B).

### Structural MRI predictors of emotional arousal

Stepwise regression identified three predictors of SAM arousal scores during negative emotion (*R*² = 0.277, *F*(3,41) = 4.431, *p* = 0.042): lentiform nucleus damage (*β* = 0.361, *t* = 2.53, *p* = 0.016); cerebellar atrophy (*β* = 0.300, *t* = 2.11, *p* = 0.042); and frontal atrophy (*β* = -0.386, *t* = -2.65, *p* = 0.012), (Table 3).

**Table 3:** MRI predictors of negative emotional arousal

Predictor	*β*	*t*-Value	*p*-Value	95% CI
Lentiform nucleus	0.361	2.53	0.016^a^	[0.242, 2.201]
Cerebellar atrophy	0.300	2.11	0.042^a^	[0.026, 1.315]
Frontal atrophy	-0.386	-2.65	0.012^a^	[-1.614, -0.217]

^a^:*P* < 0.05 indicates a statistically significant difference

### Correlation between tremor amplitude and clinical variables

Spearman analysis revealed a positive correlation between negative emotion tremor amplitude and BIDQ7 scores (avoidance) (*ρ* = 0.34, *p* = 0.022) and between negative emotion tremor amplitude and FER reaction time (*ρ* = 0.386, *p* = 0.009).

## Discussion

This study provides the first multimodal evidence linking emotional processing abnormalities to tremor variability in WD patients through EEG microstate dynamics and structural neuroimaging. Unlike prior work that focused solely on copper-induced motor circuit damage, our study uniquely demonstrates that: (1) negative emotional bias amplifies tremor amplitude via salience network hyperactivity (microstate C); (2) lentiform nucleus-cerebellar-frontal circuit damage disrupts emotion‒motor integration; and (3) body image distress mediates social avoidance behaviors — findings that have not been previously reported in WD.

### Emotion-tremor coupling: a salience network-driven mechanism

The heightened tremor amplitude observed during negative emotional states (3.79 *px* vs. 3.16 *px*, *p* = 0.012) is consistent with the hypothesis that threat vigilance is related to motor symptom severity [8]. While microstate C reflects salience network hyperactivity linked to emotion–tremor coupling, microstate A — associated with auditory and internal processing — has also been linked to anxiety symptoms in clinical populations [25]. Notably, microstate C — a neurophysiological marker of the salience network, encompassing the anterior insula and anterior cingulate — exhibited increased prevalence (16% vs. 13%, *p* < 0.001) and was correlated with both negative facial selections (*ρ* = 0.338) and tremor severity (*ρ* = 0.319). Previous studies have demonstrated that increased activity in the anterior insula and anterior cingulate during emotional stimulation has been demonstrated [26], suggesting a possible feedback loop in which tremor-related proprioceptive signals (via spinocerebellar pathways) are associated with emotional arousal, which may relate to motor control instability [27]. Recent findings indicate that negative emotions are associated with prefrontal gamma-aminobutyric acid (GABA) concentrations [28], which play crucial roles in regulating anxiety and depression. In WD, copper accumulation in the lentiform nucleus may impair GABAergic inhibition within basal ganglia-thalamocortical loops, thereby inducing more pronounced tremor variability in response to emotional perturbations.

### Structural substrates of emotion-motor dysfunction

The triad of lentiform nucleus damage, cerebellar atrophy, and frontal degeneration accounted for 27.7% of the variance in emotional arousal (*p* = 0.042), underscoring their synergistic roles: (1) The lentiform nucleus is the primary site of copper deposition in WD [29], and its damage disrupts the striatal circuits that regulate emotional salience when it is damaged. This disruption can also result in motor symptoms such as tone disorders and tremors, potentially disinhibiting limbic inputs to motor regions, similar to models of functional dystonia [30]. (2) Cerebellar atrophy undermines predictive motor control [31], thereby impairing adaptive responses to emotion-induced tremors. Previous research has identified cerebellar-limbic connections as crucial for emotion‒action coupling [7], a pathway likely disrupted.

in WD that exacerbates emotional disturbances. (3) Frontal atrophy was negatively correlated with SAM arousal (*β* = -0.386, *p* = 0.012), suggesting a compensatory suppression of emotional responses — an adaptive mechanism observed in Parkinson’s disease patients with apathy [32]. This may result in social challenges for apathetic patients owing to atypical emotional expression, thereby increasing the likelihood of social avoidance.

### Clinical implications: from mechanisms to interventions

The strong correlation between microstate C activity and tremor amplitude (*ρ* = 0.319) positions EEG microstates as a non-invasive biomarker for emotion-tremor monitoring. This finding clinically supports the following applications: (1) Targeted neuromodulation: EEG microstates are non-invasive visual indicators that are easy to use clinically and can indicate the connection between human emotion and movement. Transcranial magnetic stimulation (TMS) over the right anterior insula (salience network hub) can normalize microstate C dynamics, as shown in depression trials [33]. (2) Cognitive-behavioral therapy (CBT): Our results indicate that most WD patients suffer from negative body image. Therefore, addressing body image distress (BIDQ scores) may reduce social avoidance, indirectly stabilizing tremor by lowering emotional arousal. (3) Early intervention: Patients with lentiform nucleus or cerebellar atrophy on MRI should undergo routine emotion-processing assessments and interventions to prevent tremor exacerbation, suggesting that psychological care should receive more attention in clinical practice.

## Methodological contributions and limitations

This study bridges the gap between embodied cognition theory and systems neuroscience by quantifying tremor as a dynamic phenotype of “embodied emotion”. However, several limitations warrant caution: (1) The cross-sectional design precludes causal inferences about the relationship between emotion and tremor; longitudinal studies that track microstate changes during copper chelation therapy are necessary. (2) Sample heterogeneity was present, and subgroup analyses (e.g., hepatic versus neurological WD subtypes) were not conducted. (3) The semiquantitative nature of 1.5T visual rating scales limits structural precision; future studies should adopt high-field MRI with volumetric or morphometric analyses.

## Future directions

(1) Multimodal integration: combining fMRI and EEG microstates to map salience network–spinal cord functional connectivity during emotion induction. (2) Digital phenotyping: developing smartphone apps to capture real-world tremor–emotion interactions via accelerometry and ecological momentary assessment. (3) Mechanistic trials: testing whether TMS over the anterior insula reduces microstate C prevalence and tremor amplitude.

## Conclusion

In WD, emotional activation is associated with tremor variability, and salience network hyperactivity along with lentiform nucleus-cerebellar-frontal circuit disruption may play a role in this relationship. EEG microstate C has emerged as a promising biomarker for personalized neuromodulation, while BIDQ scores highlight the need for integrated psychological care. By combining dynamic systems theory with clinical neurophysiology, this work advances WD management beyond copper-centric approaches to embrace emotion-motor network plasticity.

## Data Availability

The data used and/or analyzed in the current study are available from the corresponding author on reasonable request.

## Abbreviations

BIDQ: Body Image Disturbance Questionnaire
CBT: Cognitive-behavioral therapy
CFAPS: Chinese Facial Affective Picture System
CSTC: Cortico-striato-thalamocortical
EEG: Electroencephalography / Electroencephalogram
EMG: Electromyography
EOG: Electrooculography
FER: Facial Expression Recognition
FTM-TRS: Fahn-Tolosa-Marin Tremor Rating Scale
GABA: Gamma-aminobutyric acid
HC: Healthy control
ICA: Independent component analysis
IQR: Interquartile range
MRI: Magnetic resonance imaging
RMS: Root mean square
SAM: Self-Assessment Manikin
SEED: SJTU Emotion EEG Dataset
TMS: Transcranial magnetic stimulation
WD: Wilson’s disease
